# Trust, confidentiality, and the acceptability of sharing HIV-related patient data: lessons learned from a mixed methods study about Health Information Exchanges

**DOI:** 10.1186/1748-5908-7-34

**Published:** 2012-04-19

**Authors:** Andre Maiorana, Wayne T Steward, Kimberly A Koester, Charles Pearson, Starley B Shade, Deepalika Chakravarty, Janet J Myers

**Affiliations:** 1 Center for AIDS Prevention Studies, AIDS Policy Research Center, University of California, 50 Beale St, suite 1300, San Francisco, CA 94105, USA

**Keywords:** Trust, Confidentiality, Acceptability, Health information exchanges, HIV, Patient data-sharing

## Abstract

****Background**:**

Concerns about the confidentiality of personal health information have been identified as a potential obstacle to implementation of Health Information Exchanges (HIEs). Considering the stigma and confidentiality issues historically associated with human immunodeficiency virus (HIV) disease, we examine how trust—in technology, processes, and people—influenced the acceptability of data sharing among stakeholders prior to implementation of six HIEs intended to improve HIV care in parts of the United States. Our analyses identify the kinds of concerns expressed by stakeholders about electronic data sharing and focus on the factors that ultimately facilitated acceptability of the new exchanges.

****Methods**:**

We conducted 549 surveys with patients and 66 semi-structured interviews with providers and other stakeholders prior to implementation of the HIEs to assess concerns about confidentiality in the electronic sharing of patient data. The patient quantitative data were analyzed using SAS 9.2 to yield sample descriptive statistics. The analysis of the qualitative interviews with providers and other stakeholders followed an open-coding process, and convergent and divergent perspectives emerging from those data were examined within and across the HIEs.

****Results**:**

We found widespread acceptability for electronic sharing of HIV-related patient data through HIEs. This acceptability appeared to be driven by growing comfort with information technologies, confidence in the security protocols utilized to protect data, trust in the providers and institutions who use the technologies, belief in the benefits to the patients, and awareness that electronic exchange represents an enhancement of data sharing already taking place by other means. HIE acceptability depended both on preexisting trust among patients, providers, and institutions and on building consensus and trust in the HIEs as part of preparation for implementation. The process of HIE development also resulted in forging shared vision among institutions.

****Conclusions**:**

Patients and providers are willing to accept the electronic sharing of HIV patient data to improve care for a disease historically seen as highly stigmatized. Acceptability depends on the effort expended to understand and address potential concerns related to data sharing and confidentiality, and on the trust established among stakeholders in terms of the nature of the systems and how they will be used.

## **Background**

### **Health information exchange (HIE) systems**

Health information technology (HIT) has been identified as a critical tool for improving medical care and treatment while holding down costs [1-3]. HIT has the potential to improve the quality and efficiency of medical care through more informed decision making, enhanced communication with patients, better tracking of clinical indicators and medical records, and improved management of databases about diseases and treatments [1,3-9]. Within the United States (US), HIT has become an essential component of efforts to reform the healthcare system, including support within the American Recovery and Reinvestment Act of 2009 for the adoption of electronic medical records and within the Health Information Technology for Economic and Clinical Health Act to aid in the development of a nationwide HIT infrastructure [10].

In order to facilitate information flow across the healthcare system, provider groups, hospitals, insurers, and governments are taking steps to implement HIEs, which are characterized by formal agreements and technologies that facilitate the electronic movement of health-related information across organizations within an area or community [11]. Despite their promise, there are significant barriers to widespread adoption of health information technology and exchange. Recent research has shown a relatively low uptake of electronic records systems in both private provider and hospital settings [12,13]. Among the obstacles are financial and personnel investments [14], development and adherence to common industry-wide protocols to facilitate data transfers among systems [15,16], and concerns about the confidentiality of personal health information [5,17-20].

### **Trust in HIEs and confidentiality of electronic data sharing**

Trust, in general, implies certain vulnerability and the belief or expectation that actions or social relationships will cause no harm and/or will provide a benefit [21]. Not surprisingly, trust is a key factor in the delivery of healthcare, as research has shown that high levels of provider/patient trust is conducive to more effective healthcare [22]. Consequently, the building of trust in healthcare settings has emerged as a central concern [23-25]. Trust in healthcare can be seen as a three-part relationship between patient (truster), provider or organization (trustee), and the specific context of delivering healthcare. In that context, trust also relates to the expectation that an entity will adhere to their fiduciary obligations [26].

According to Heuwinkel and Deiters, trust factors and attitudes related to acceptance of HIEs operate both at an objective and a subjective level and are influenced by the interplay of technological, sociological and psychological issues. On the one hand, users need to trust in the technological elements of the exchange system and have confidence in the objective security measures in place to protect personal data. On the other hand, users need to assign a value and expect a benefit from the exchange system. Users make determinations about the value of electronic data sharing within a larger social and psychological context that affects how they navigate mutual interactions and calculate benefits and potential risks of their choices [27]. Building a ‘framework of trust’ among the different entities participating in a HIE related to the privacy and confidentiality of data sharing [28], and adherence to a ‘common framework’ that includes basic policies and standards have been proposed to overcome challenges to HIE implementation [29].

Early research documented substantive concerns about the security of personal health information stored in electronic systems [30-34]. However, recent studies suggest a growing acceptance among healthcare providers and patients of health technology [35,36], and greater willingness to share information through HIEs [37,38]. Because such acceptance is enhanced when patients and providers are informed or involved in a system’s development [39-41], experts emphasize the importance of considering attitudinal issues, such as buy in and trust from stakeholders, including patients, when developing new HIEs [42,43]. Different theoretical constructs, including several variations of the Technology Acceptance Model (TAM) [44], have been used to understand what is necessary for the successful implementation and acceptability of technological systems. Tung *et al.* propose to add trust as another dimension to the TAM model influencing beliefs and attitudes, intention, and perceived usefulness to use [45]. However, while trust in electronic systems’ privacy and security measures, if accompanied by oversight and stronger accountability mechanisms, could be a facilitator to HIE implementation and adoption [42,46], there is limited research on the impact of trust and how attitudes toward privacy and confidentiality may influence the development and implementation of HIEs and the integration of information technologies with human immunodeficiency virus (HIV) care.

### **Purpose of this analysis**

HIV care is an important but challenging sector for the rollout of information technologies. Given the complexities of treating HIV disease, information exchange would be expected to be useful for improving care outcomes and delivering multiple services. New information technologies have already demonstrated success in HIV care settings [47], including improved care coordination [48] and enhanced provider satisfaction [49], as well as increased acceptability among patients [50]. However, the relatively high levels of stigma toward the disease have greatly heightened confidentiality concerns around HIV [51], resulting in HIV-related patient data historically enjoying greater legal protections than other kinds of medical information [52].

Considering the stigma and confidentiality factors historically associated with HIV disease, we examine in this article how trust—in technological systems, operational procedures, and people—influenced the acceptability of data sharing among patients, providers, and other stakeholders before implementation of six distinct HIEs in different regions around the US. Our analyses identify the concerns expressed by stakeholders about the confidentiality of electronically shared data and focus on the factors that ultimately facilitated acceptability of the HIEs.

## **Methods**

The Health Resources and Services Administration HIV/AIDS Bureau (HRSA/HAB) sponsored a four-year Special Project of National Significance (SPNS) known as the Electronic Networks of Care Initiative. It consisted of one cross-site evaluation center at the University of California San Francisco (UCSF) and six unique demonstration sites independent from each other implementing HIEs tailored to the local HIV epidemic and the care systems in those locations. These HIEs were designed to enhance HIV care, promote the flow of HIV-related health information across clinical settings, community-based organizations, and public health agencies, as well as to improve patients’ access to their health records. For that purpose, each HIE created different kinds of electronic platforms to share patient data among the collaborating medical settings and outside agencies. Table 1 presents the key characteristics of the six HIEs.

**Table 1:** Description of the six Health Information Exchanges part of the Electronic Networks of Care Initiative

**Site**	**Collaborating Institutions**	**Users**	**Goals and Scope of the HIE**	**Description of Information Exchanged**
**Bronx-Lebanon Hospital Center**	- Hospital based HIV specialty clinic	- Clinical providers	Clinic-based system to improve coordination of care and health outcomes	· Bi-directional limited in real-time information exchange
				· Medical providers have access to reminders on clinical tasks/priorities and patient clinical indicators are exchanged with case management and medical support service agencies.
	- Support services	- Support services (case managers, medical social workers, nurses)		
City of Paterson*	- Primary care clinics	- Clinical providers	Localized area newtwork of care to exchange information and quality tools to enhance efficiency, continuity, and quality of HIV care	· Patient information is exchanged through a Web-based system in real time among administrators, medical providers and staff in primary care clinics, support service agencies, and HIV testing sites for quality assurance and to improve quality of care and patient satisfaction.
	- Support services	- Clinic staff		
	-Testing sites	-Administrators		
Duke University*	- Hospital based primary care clinic	- Clinical providers	Localized area newtwork of care to enhance efficiency, continuity, quality and delivery of HIV care among partner agencies within a RHIO	· Information uploaded and shared through a regional server
				· Patient clinical indicators are exchanged among a medical setting and case management support service agencies.
	- Support services	- Support services (case managers and social workers		
		Administrators		
**LSU Health Care Services Division**	- Emergency departments, - Outpatient and inpatient clinics in 7 public hospitals	- Clinical providers	Public health exchange to improve HIV case reporting, identify and link to care HIV + individuals out of care or lost to care	· Real-time, bidirectional information exchange between a public health jurisdiction and a health care delivery system
	- Office of Public Health	- Surveillance and public health personnel		
				· Alerts are exchanged to identify out of care HIV infected individuals who seek treatment for other conditions and link them to HIV care.
	- Louisiana Public Health Institute	-Administrators		
New York- Presbyterian Hospital*	- HIV care providers - Support services - Health Insurer	- Patients - Clinical providers - Support services Administrators	Localized area newtwork of care to improve coordination and increase quality of care	· Web-based system
				· Provider and patient portals allow patients access to their own information, and allow medical providers, administrators, and social workers, case managers, and outreach workers from support service agencies to exchange patient information.
**St. Mary Medical Center Foundation**	- Primary care clinic	- Clinical providers	Clinic-based to improve patient quality and efficiency of care	· EMR’s interoperable, bidirectional exchange
	- Laboratory	- Clinic staff		· Patient laboratory requisitions and results as well as medication prescriptions and refills are exchanged among clinic, laboratory, and pharmacy staff to avoid duplication of services and facilitate communication
	- Pharmacy	- Pharmacies		
		- Laboratories		

* Involved in fiduciary relationship among partners.

### **Data collection**

We present quantitative and qualitative data collected at each of the six sites prior to HIE implementation. Within health services research, a mixed methods approach that integrates qualitative and quantitative data can help to examine the complexity of the healthcare environment, health-related issues, measure outcomes, and evaluate interventions [53]. The data were collected between August 2008 and April 2010 as part of the UCSF multi-site evaluation. The study was reviewed and approved by the Institutional Review Board (IRB) at UCSF and by the corresponding IRBs at the six implementing sites.

### **Quantitative surveys**

We administered a quantitative survey to HIV positive patients at each site to obtain a description of the patient population and measure their willingness to share information using HIE. A convenience sample of patients (n = 549) was recruited by site staff at HIV clinical care settings involved with each HIE. These patients were administered an Audio Computer-Assisted Self-Interview (ACASI) survey, which included questions about demographic information and HIV medical care. In addition, the patients were asked to agree or disagree with a series of statements indicating willingness to have health information electronically shared with various providers, payers, and agencies: their primary HIV care provider; other clinicians in the clinic of the primary HIV care provider; other non-clinical staff in the clinic of the primary HIV care provider; non-HIV specialists (*e.g.*, cardiologists, ob/gyn); other healthcare providers (*e.g.*, emergency or hospital personnel); pharmacists; HIV support service organizations (*e.g.*, case managers); other non-HIV-specific support service organizations (*e.g.*, drug treatment programs, mental health programs); private health insurers, government health insurers; and local health departments. For example, two specific survey items were ‘I am willing to allow my personal health information to be shared with my primary HIV care provider (*e.g.*, physician, nurse practitioner, physician assistant) using a secure electronic network,’ and ‘I am willing to allow my personal health information to be shared with my private health insurers using a secure electronic network.’ Responses were recorded on a five-point scale ranging from strong disagreement (0) to strong agreement (4).

### **Qualitative interviews**

UCSF investigators experienced in qualitative research conducted the interviews either face-to-face during site visits or over the phone. We conducted a total of 66 semi-structured interviews with three types of key informant stakeholders: project staff and information technology (IT) specialists associated with the demonstration site project (n = 22); staff from community-based organizations and public health agencies collaborating in the design and implementation of the HIEs (n = 12); and intended users of each HIE (n = 32), such as medical providers and staff in clinical settings, as well as social workers, case managers, and medical social workers at agencies external to the medical settings but linked to a HIE. To ensure diversity of perspectives, we collaborated with site staff to identify stakeholders to be included in our sample. The sample size at each site varied according to the project and the number of stakeholders involved in each of the HIEs.

Using different semi-structured guides parallel in content for each of the three types of key informant stakeholders mentioned above, the interviews focused on understanding the development of the HIE systems, the planning process and preparatory work, and the expected benefits, as well as the technological, attitudinal, and structural barriers and facilitators to acceptability of data sharing, including issues related to trust and confidentiality. We specifically asked all stakeholders what confidentiality-related issues they expected or had had to address, including their own concerns as well as those of the patients. Probes were used to prompt information that was not spontaneously offered by the participants and further enquire into topics of interest. For HIE system users, we specifically conducted interviews after they had been trained on how to use the systems, but before those systems were implemented. All interviews were audio-recorded and transcribed.

### **Data analyses**

We initially analyzed quantitative and qualitative data separately. Subsequently, the two datasets were compared, with the quantitative findings from the patient survey helping to inform and better frame qualitative findings from providers and other stakeholders.

### **Quantitative data**

We cleaned the data as needed and generated descriptive statistics for the sample. Following this, the mean levels of agreement and the corresponding 95 % confidence limits for each of the questions about willingness to share medical information via HIE were calculated. All analyses were conducted using SAS V9.2.

### **Qualitative data**

All interviews transcripts were entered into Atlas ti©, a software program designed to organize qualitative data and to facilitate analysis. Data analysis procedures followed an open-coding process developed by Strauss and Corbin [54]. During the initial phase of analysis, three analysts individually read a subset of interviews and developed preliminary codes based on domains from the interview guides and emerging concepts and categories. The analysts refined those preliminary codes and discrepancies in coding were solved during team meetings. The final version of the codebook consisted of 16 coding topics, which the analysts then applied across all the interviews. Each interview was coded by a primary analyst and verified by a secondary analyst. Coded data were summarized within each of the six sites. Convergent and divergent perspectives were then examined within and across sites. Analyses for this article included all data associated with the codes ‘confidentiality,’ ‘process of collaboration,’ ‘challenges,’ and ‘expected benefits.’ The rest of the codes, while relevant for the overall evaluation of the HIEs, did not apply to trust or confidentiality issues, and are thus not considered here.

## **Results**

We first present quantitative data reflecting on the patients’ willingness to share personal health information electronically. We then present findings from the qualitative interviews with providers and other stakeholders on their views and acceptability of data sharing through HIEs.

### **Quantitative data from patients**

**Figure 1:**
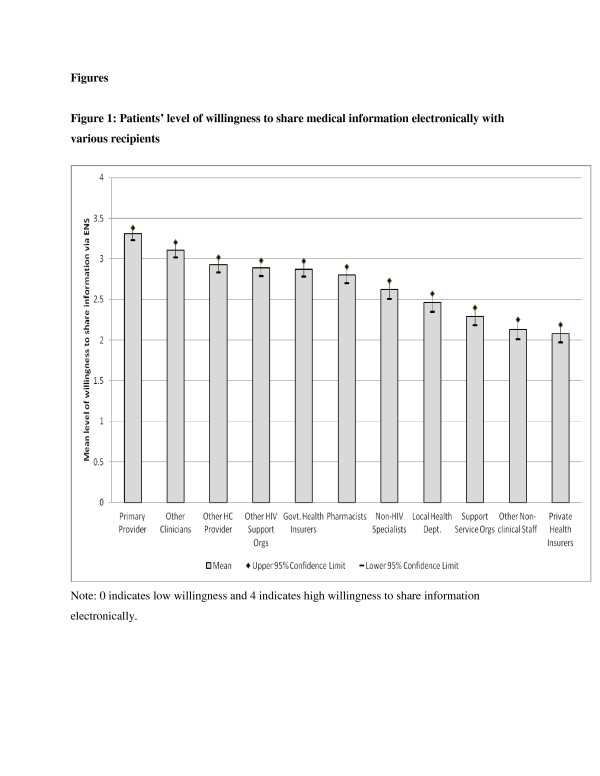
**Patients’ level of willingness to share medical information electronically with various recipients.** Note: 0 indicates low willingness and 4 indicates high willingness to share information electronically.

As reflected in Table 2, the 549 patients in our sample were diverse in terms of race, ethnicity, gender, sexual orientation, education, and risk factors for HIV. Figure 1 displays the mean levels and the corresponding 95 % confidence limits of patient willingness to share medical information electronically with different types of providers, payers, and institutions. Patients were most comfortable sharing health information with their primary care provider, followed by other clinicians, other healthcare providers, HIV support organizations, and government health insurers. At the other end of the spectrum, patients were less willing to share health information with private health insurers and other non-clinical staff. The mean levels of willingness in every category fell above the mid-point of the measurement scale (which was defined as the point where patients neither agreed nor disagreed with sharing information), reflecting an overall comfort with electronically sharing information.

**Table 2:** Descriptive characteristics of the patients in the quantitative sample (N = 549)

**Characteristics**	**Mean**	**95 % Confidence Interval**
Age (yrs.)	45.3	44.5, 46.1
	**n**	**%**
Gender		
Male	347	63.2
Female	197	35.9
Transgender	5	0.9
Race / Ethnicity		
Caucasian	113	20.6
African-American	285	51.9
Hispanic / Latino	106	19.3
Mixed	29	5.3
Other	16	2.9
Education		
Less than high school	179	32.6
High school or G.E.D	240	43.7
Greater than high school	130	23.7
Annual Income		
No income	127	23.1
Less than $5000	121	22.0
Between $5000 and $10000	165	30.1
Between $10000 and $20000	87	15.9
Greater than $20000	49	8.9
Sexual Orientation		
Heterosexual	320	58.1
Homosexual	156	28.4
Other	73	13.3
Risk Factors for HIV		
MSM	186	33.9
IDU	63	11.5
Heterosexual contact	192	35.0
Other	108	19.7
Homeless	120	21.9

### **Qualitative data from project staff, IT specialists, and providers**

These findings are organized to present stakeholders’s perspectives at four different levels: their views and expectations about patients’ acceptability of data-sharing; their own views regarding acceptability of patient data sharing; their views and experiences that reflect upon acceptability at an institutional level; and trust as an overarching factor influencing the stakeholders’ views on data sharing. Table 3 shows the main factors influencing acceptability included in the qualitative findings.

**Table 3:** Main factors influencing stakeholders’ acceptability of electronic data sharing

**Different Levels of Stakeholder Views**	**Factors Influencing Acceptability of Data Sharing**
Stakeholder views on patient acceptability	· patient familiarity with electronic technology
	· patient trust in institutions and staff
	· expected benefits for patients
Stakeholder views on stakeholder acceptability	· trust in the security systems to protect data
	· greater understanding of extent of information sharing
	· greater understanding of parameters of access by differing individuals
Stakeholder views on confidentiality and acceptability at the institutional level	· establishment of legal foundation for data sharing and contractual agreements
	· trust in system security
	· trust in HIE agencies and staff, partially dependent on agency precedents for sharing electronic data sharing
Importance of trust in establishing acceptability	· trust between patients and providers
	· trust between different stakeholders and between agencies
	· trust in system security

### **Patients see HIEs as a change in the mechanism but not in the practice of exchanging data**

In general, stakeholders anticipated no major concerns from patients about sharing data. The consensus was that most patients would be willing to allow their health information to be shared electronically. Those perceptions correspond with the patients’ willingness to share data shown in the quantitative data. Stakeholders identified familiarity with the use of electronic technology, trust in the institutions and in the staff providing services, and the expected benefit of HIE as facilitators of the acceptability of data sharing among patients.

Stakeholders identified a variety of factors that they thought contributed to overall patient comfort with data sharing. One of the primary factors was the growing acceptance of health-related IT, a result of the increasing use of electronic records systems in clinical settings. Patients have become used to seeing their information reviewed in or entered into a computer while receiving services:

"‘And again regarding electronic mistrust issues.... we’ve kind of progressed over the past five or six years to using our electronic, internal electronic medical record system a lot more and so patients are very used to seeing people typing… as soon as you show up things are going into computers and… all prescriptions are electronic and we usually are using the computers while we’re talking to people so my guess is that some of that’s not as much of an issue as it has been in the past.’ —Medical Director"

Stakeholders also reported that, to patients, HIEs represent a change only in the mechanism but not in the practice of exchanging data. Stakeholders spoke of how patients knew that their information was already shared by fax, phone, or paper, and that this awareness contributed to the acceptability of electronic exchange of information. Similarly, familiarity among patients with the concept of having to sign a release of information in order for their information to be shared with external medical providers or support agencies also facilitated acceptability of electronic data sharing:

"‘I guess because of the way I’ve shared it with the clients…, to them it’s just another way…another technology....we already share it anyway…. it’s the same thing as me calling the doctor’s office saying can you give it to me, as me pulling it up in the system. I think they view it like we already know, …we’re going to know anyway. … it’s hard for me to service you as a client when I don’t know what’s going on with you medically. So that helps me to give you what you need and it helps it go over a whole lot faster. So they don’t look at is as a threat of ‘my information’s going to be given out’ where ‘it’s my case manager and it’s my doctor so it’s cool” —Administrator and Case Manager."

Stakeholders emphasized that patients’ acceptance of HIE could be facilitated by being transparent about a system’s purpose, the types of information to be exchanged, the expected benefits, and the individuals and/or agencies that would have access. One case manager anticipated that, once the local HIE went live, a line would be included on the release of information forms so that clients would know that health information from the medical setting would be electronically available to support services agencies. Rather than encountering opposition to electronic data sharing through statements such as ‘well, I don’t want you to know my business,’ or ‘how dare you be able to do that,’ the same case manager expected clients to ask, ‘who else has access to this information,’ and to help her clients ‘…understand that we’re on this system together to assure quality of care more than anything else.’

Stakeholders reported that sites had conducted formative work pre-development of HIEs and consulted patients regarding their views on electronic data sharing. In one site, stakeholders stated that needs assessments had shown that patients thought information was already being shared with other agencies in an effort to provide better care. Other sites, also taking into account the opinions of patients, took a number of steps to control access to electronic health information, with greater degrees of access generally being reserved for personnel most directly involved in clinical care. For example, at one site, a decision was made not to give clerical staff access to detailed clinical information in electronic reminders after a patient advisory board raised concerns about privacy. In another site, clerical staff registering patients were given alerts that only told them that patients needed to talk to a medical provider about further testing, without specifying what kind of testing was needed. And at a third site, stakeholders reported that patients only became comfortable with the idea of information being exchanged among partner agencies once they knew that unique identifiers and not names would be used in the system.

Nevertheless, a few of the users, based on their direct experience working with patients, noted that a small number of ‘cautious’ patients might either object to or would need additional reassurance before they agreed to data-sharing. According to them, such patients were likely to include older African Americans, substance users, undocumented Latinos and other groups that may be more suspicious of the healthcare system. In addition, objections were anticipated from patients who are very secretive about their HIV status or were categorized as ‘hard to work with.’ Related to this issue, a clinic coordinator stated, ‘There are a few patients who are just very cautious and concerned about their health information and...[their] needs are addressed on a one-to-one basis ...but as a rule it’s not something that we deal with on a day to day basis.’

### **Project staff and users’ trust in the systems and the security of the systems**

Stakeholders were willing, and in many cases enthusiastic, to share patient data electronically because they expected that it would make their work more efficient and be of benefit to patients or clients. Their acceptance of data sharing was facilitated by trusting the systems and the security measures in place to protect the data, as well as by learning about the extent of the information to be shared and who would have access to the information exchanged. Their perspectives were influenced not only by knowledge about technological protections of data (*e.g.*, encryption), but also by attitudes about the appropriate extent of information to be shared and the types of individuals who would have access

For the most part, their potential concerns about confidentiality had been resolved during the HIE design, development, and training prior to our interviews. Stakeholders reported that their approaches to addressing those concerns depended on the scope of the project, collaborative processes among HIE partners, and the needs assessments the sites had conducted when designing their systems. For example, one site conducted a comprehensive legal and ethical review to determine what information could be exchanged and with whom. A collaborator part of that review stated:

"‘And I can summarize everything that I’ve been doing as falling into the category of trying to maximize the information sharing that takes place and maximizing the benefits to everyone…while still observing any applicable privacy restrictions, which would come from state law or federal law or any other applicable standard….I’m trying to help them get the most out of this, you know, without legally violating anyone’s privacy.’"

That review helped to alleviate stakeholder concerns around confidentiality by establishing clear legal boundaries for the project’s scope and types of information to be exchanged. The formative work with patients pre-development of HIEs mentioned earlier also helped alleviate stakeholders’ concerns regarding electronic data sharing. In order to feel like concerns about confidentiality had been addressed, stakeholders needed to have a clear understanding of a HIE’s purpose and intended uses, and the security measures to protect patient data. For example, our interviewees explained that stakeholders wanted to know about the access rights to a HIE, such as who would be able to log in and how, and the extent of the information to be exchanged. Such knowledge helped assuage stakeholders’ concerns and contributed to their personal buy-in to the system. That knowledge also equipped them with information they could use to educate patients about the HIE. Furthermore, as stated by project staff, users’ buy-in was important because the trust of the users in the system will reflect in how they explain it to the patients, thus influencing the patients’ trust in the HIE, ‘because part of what we do is we help case managers really feel like this is doing what’s best and I think that translates to the patients; that trust translates.’

Paralleling expectations about patient attitudes, stakeholders’ personal acceptance of the HIEs was also influenced by the perception that they represented only one step beyond ‘business as usual,’ and sharing information on paper or by phone. For example, one medical provider noted that electronic exchange simply provides a different mode of communication but that the fundamentals of sharing clinical information remained the same. Stakeholders pointed out that, at all agencies, procedures already existed to ensure patient privacy. Many partner agencies within the HIEs already were reporting either identifiable or de-identified patient information to local medical consortia, to state or local health departments for surveillance purposes, or to funding agencies as a condition of grants that supported clinical operations. One medical provider pointed out that the problems that may lead to unintended lapses in confidentiality (*e.g.*, human error) could occur both with electronic or non-electronic exchange of healthcare information:

"‘...I’m sure there are ways that somebody could get into the computer system and get confidential medical records. In faxing prescriptions things could probably be accidentally faxed to the wrong fax number**….’** —Medical Provider"

### **Acceptability of data sharing at an institutional level**

Acceptability of data sharing involved overcoming barriers about privacy issues and potential liability for the institutions connected with the HIEs. Institutions’ acceptance was facilitated by reviews regarding the legal aspects of data sharing for the institutions, as well as by trust in the security of the systems, trust in the staff and/or agencies involved in the HIEs, and whether there was a precedent within the institutions sharing data electronically.

A number of stakeholders, particularly project and IT staff, reported having spent considerable time and energy addressing confidentiality issues raised by administrators, legal affairs departments, and other entities within institutions sharing electronic information. Some spoke of the challenge of having to overcome the resistance, underlying mistrust and ‘aversion’ to sharing data among players distal to the development of the systems, even when that sharing was legitimate, lawful, and secure. The process seemed to be the most cumbersome and prolonged when academic and governmental agencies were involved.

The process for overcoming institutional concerns about confidentiality and liability included drafting, reviewing, re-drafting, and finally approving data-sharing legal contractual agreements between collaborating agencies. Those agreements had to address myriad issues, including which institution would house the servers for the HIE, technical discrepancies between IT groups at participating agencies, and the types of information to be exchanged. In some cases, the process involved clarifying the legal parameters of HIPAA, including what kinds of data sharing were forbidden by HIPAA, but also the kinds of data sharing activities that were permissible without patient consent. For example, at one site, project staff had to work with participating institutions to clarify that, under HIPAA, a healthcare plan payer could receive patient data because it was part of the operations necessary to coordinate care:

"‘The case...needs to be driven home to them that really you can’t use HIPAA to say ‘We can’t give you that information because of HIPAA..’ HIPAA also provides what’s allowed to be shared and under what circumstances it’s allowed to be shared. And there seems to be an education issue for a lot of organizations that are providing care but try to use HIPAA... as a weapon to not share data when in actuality they can and they’re allowed to. But they always fall back on, ‘We can’t tell you that because of HIPAA.” —Project Director"

Stakeholders reported that appeasing concerns of administrators and legal affairs officers often involved demonstrating that the operations of the HIE would not ultimately reflect poorly on the larger institution. Although those formal reviews were arduous, they were successful at assuaging potential concerns and allowing projects to move forward. Similarly, the use of ‘off the shelf’ HIE systems that had been fully vetted by other organizations helped facilitate reviews and approvals at some of the sites:

"‘...we had to go through a privacy analysis with [name of company]...; and they have their own lawyers and most everything that we needed was already written up for us because ...they have a million hospitals across the country. But...for the most part patient privacy issues for us aren’t as much of an issue because we are already dealing with that every day. So...we were able to determine…that the data was encrypted, that [the system] has been vetted and approved by all of the different boards of everybody...’ —IT staff"

### **The importance of trust factors in the acceptability of HIEs**

Trust in the confidentiality and security of electronic data sharing, as well as existing trust among the different stakeholders, had an overarching influence in the acceptability of the systems. Stakeholders’ personal attitudes, expectations about patient perspectives, and experiences with institutions mutually reinforced one another. The importance of establishing and maintaining trust between patients and providers and between stakeholders from different institutions was repeatedly mentioned by our interviewees as an issue influencing the acceptability of the HIEs. As already noted, a number of HIE users stated that patient comfort with the HIEs was forged in part by receiving information from the providers. In other cases, stakeholders reflected on how patient trust of the new HIEs was, in a sense, an extension of the trust and satisfaction they felt toward their personal providers and the institutions in which they received care:

"‘I think most of our patients feel like their information is safe. They expect, because of the nature of coming to a clinic, that it’s based in an academic research institution that there will be information sharing within the scope of treatment and coordination of care and that we will be measuring quality data on them…our patient population at least - is a very trusting population. They trust that we’ll keep their information safe.’ —Clinic Coordinator"

Similarly, an administrator at a case management agency mainly serving an ethnic minority referred to the existing trust that clients have in the agency as overcoming privacy concerns they may otherwise have had:

"‘I think in the communities that we serve there are tremendous concerns about privacy, tremendous... bias towards secrecy. Even just as a precaution.... But because most of our staff are themselves [of the same heritage as the patients], because we have a lot of history with the community, there’s a lot of trust.’"

Somewhat different factors influenced how trust between stakeholders and institutions affected the acceptability of the HIEs. Within an information exchange system, there are multiple potential dimensions related to trust. Some operate at a within-institution level (*e.g.*, between an individual provider and the institution where he/she work), and others operate at a between-institutions level (*e.g.*, between stakeholders from different institutions). In our interviews, the more salient dimension was the latter (between institutions), a finding that is likely reflective of the HIE’s fundamental purpose, which is to facilitate interactions across institutional boundaries.

In general, stakeholders from participating agencies indicated that they trusted the staff and/or the leading organization that had taken primary responsibility for developing each HIE. In most cases, that entity was either a university and its affiliated hospital or a public health institution. This confidence extended to the organizing entity’s staff (*e.g.*, technical advisors, IT specialists, and lawyers) and to the systems infrastructure and security protocols that had been established. As illustrated by the quote below, trust of the developing partner was particularly salient in interviews conducted at collaborating agencies that were small in size or did not have their own IT systems in place:

"‘This chief IT person has been involved in this [name of the HIE] for a long time. He said ‘we’ve done this with big organizations; this is what we do. ‘.... And the community organizations feel very trusting. I mean they have this big lawyer IT person coming in saying this is what you have to do. They’ve been very trusting, much more trusting that I expected.... these small community based organizations can’t go out and hire a health information lawyer to go through and give them an audit and tell them what they need to do to protect themselves or when they don’t. So when they can leverage somebody from [name of the HIE] to come in and say ‘this is the legal way to do it’ they get that benefit without actually having to expend on it.’ —Project staff"

Across all HIEs, stakeholders indicated that their own trust—and that of the more distal players in positions of leadership—were driven heavily by confidence in the security of the systems. This made IT staff members especially key to success not only because these individuals made the systems operable, but also because their effort’s quality and thoroughness influenced other stakeholders’ opinions about a HIE’s usability and security. IT staff members’ role was further enhanced by their ability to offer technical support, a task that necessitated clear communication to explain in simple language data encryptions, virtual private network (VPN) tunnels, and firewalls. Our interviewees reported that those explanations helped build trust by assuring stakeholders and others involved in decision-making that adequate safeguards would be in place to secure patient data. On the other hand, an IT staff member noted that many well-founded concerns raised by project staff and users not ‘technically aware’ could be relatively easily addressed and explained to them, while IT staff members were more concerned about the more complex ‘under the cover’ system security issues, such as cross-site scripting holes, a vulnerability which allows attackers to bypass security mechanisms on web content by injecting malicious scripts or codes in order to gain access-privileges to sensitive information:

"‘And it’s a little bit like magic. And you hear a lot of things in the press and they’re [stakeholders] concerned with things that have already been taken care of. And the really big technical threats are things that IT staff are concerned with, we talk about those things and follow those things too. There’s always the latest exploit or something going on. And we’re trying to stay ahead of the typical things that can happen on the Internet like ....CNS poisoning and all these other things that are going on, to make sure the system can acquire whatever remedies it has to those things....for example high level people are worried about passwords....Passwords are relatively easy to solve and that has to do with access to the system.... The difference is knowing what can technically happen under the covers and seeing what is happening above the covers...you can have the right password and the right user ID and have several passwords and all those sorts of things and still fall a victim to cross site scripting, and you won’t even know it’s happening.’"

## **Discussion**

Because of the stigma still associated with HIV disease, we initially thought that confidentiality might be a key barrier to HIE in HIV care settings. Instead, we found widespread acceptability for HIEs across patients, providers, and other stakeholders. Considering both our large quantitative patient dataset and qualitative interviews with stakeholders at the six sites part of HRSA’s demonstration project, our findings have broad implications for HIV treatment settings. They suggest that those who deliver and receive HIV care have grown more comfortable with integrating HIV-related patient data into modern technologies that may improve information flow and HIV care. These attitudes appear to be driven by growing comfort with information technologies, confidence in the security protocols utilized to protect data, trust in the providers and institutions who use the technologies, belief in the benefit to the patients, and awareness that electronic exchange represents an enhancement of data sharing already taking place by other means. Our quantitative data illustrate that patients were willing to share their information electronically with recipients who may help provide them with better care, such as medical providers, HIV support organizations, and government insurers. Thus, while patients still may be concerned about stigma and about people outside their healthcare finding out about their HIV status, they may not be that concerned about sharing their information with people who may impact their healthcare. The farther the data-recipient is from impacting their health directly, such as non-clinical staff or private insurers, the less willing were the patients to share their information with them, perhaps under the logical inference that there is no reason for those people to know. Although we found broad acceptability for electronic data sharing, the stakeholders’ attitudes continued to reflect an underlying expectation that access to information would be carefully monitored. Such perspectives included trust that the data were secured by encryption and other technological controls and that access rights to the systems had been well considered.

While the factors above acted as facilitators, our data suggest that barriers for electronic data sharing at the institutional level need to be carefully considered. Because some of the HIEs were high profile endeavors likely to attract attention from outside entities (*e.g.*, media), the sites had to go through rigorous scrutiny at different institutional levels to assess their legality and potential liability. Many of the sites had to engage in long and formal reviews that thoroughly documented the design of the systems and the potential legal or public relations implications of implementing HIEs. These processes were further complicated by the very nature of the HIEs themselves, which link disparate institutions. The attitudes of institutional authorities were swayed by careful review of security procedures and governing laws. Those processes, however, were more cumbersome than the ones required to educate patients or providers. As such, the process of HIE development ultimately became one of forging shared vision and consensus among institutions.

The importance of consensus building and trust in the systems was also evidenced in the providers’ reflections on their own evolving attitudes regarding data sharing. The closer the stakeholders were to the process of HIE design and development, the greater their level of trust. System development and implementation required taking into account the attitudes and concerns of a wide variety of individuals potentially affected by a HIE. The often lengthy preparatory processes ended up serving as a crucial component of early success, because it allowed sites to address concerns, educate stakeholders about design choices, and build trust and support for system implementation. Without these processes, it is likely that stakeholder perspectives may have been very different.

HIE acceptability may depend on preexisting trust among patients, providers, and institutions. In our data, patient trust of the systems appeared to be driven in part by the confidence they already held in their providers and care facilities. It is important for further research to explore patients’ specific understanding of the data kept and currently shared in medical records [37], and their rationale behind supporting or not supporting enhanced information sharing [55]. It would be particularly relevant to examine these issues with patients from communities that are likely to still be suspicious of or fear data sharing. Although stigma against HIV itself has declined [51], legal sanctions and social norms still may influence some populations affected by the disease [56]. For example, publicly funded clinical settings provide critical safety net health coverage to undocumented workers who, because of the fear of deportation, may be far more hesitant to seek care if information about them were to be shared across regional health networks and government agencies, such as health departments.

In our findings, some users raised concerns that patients of certain ages or racial/ethnic backgrounds might object to, or might need additional reassurance, before they agreed to electronic data sharing. Our patient survey data did not permit us to test those hypotheses because the present study was not powered to detect attitudinal differences in patient subgroups. Further research will be needed to determine whether there are indeed specific groups of patients who are reluctant to share their personal health data electronically. If findings were to reveal that provider perceptions are not matched to actual racial/ethnic differences in patient attitudes about the acceptability of sharing health data, then it also would be important to identify the factors that lead to such a discrepancy.

Stakeholder perspectives were derived by the trust they placed in their collaborating HIE partners. The process of collaboration helped to leverage resources, providing opportunities for smaller agencies to benefit from the technical expertise and support provided by the IT staff affiliated with a HIE. Smaller agencies also received legal advice and health information systems capacity-building that they otherwise would not have been able to afford. In this sense, the HIEs helped to distribute resources more evenly across agencies and to enhance, access to technologies that could help address unmet needs.

Clinical environments should not anticipate easy implementation of information exchanges if there is existing distrust between patients and providers, or if partnering agencies have historically been suspicious of one another. Efforts must be made to forge common bonds before directing resources to setting up the technological infrastructures required for data exchange. Addressing attitudes on trust and confidentiality of sharing patient data electronically will influence the perceived usefulness, the perceived ease of use, and adoption of HIE. Gaining stakeholders and patients trust in the security and confidentiality of the data is absolutely essential when designing and implementing HIEs.

Our findings suggest that confidentiality concerns related to sharing data electronically will not be barriers to HIEs becoming integrated into patient care, at least among the general population of patients and providers in publicly funded clinics. Rather, the perception that the HIEs cause no harm and may in fact benefit patients and other stakeholders is likely to influence their use and acceptability. Nevertheless, the question remains whether human error or a breach in confidentiality, whether as part of the HIEs or a highly publicized event in the field of technological communication or online information exchange, may emerge to affect the expected benefits and the trust in the HIEs. Furthermore, acceptability and trust in the safety of personal information shared through HIEs may influence future policy issues, laws, and privacy rules related to a healthcare system and industry driven by new and ever changing technologies and electronic communication [57-60].

Our study has several limitations. First, the data were collected exclusively in public settings. Stakeholder attitudes may differ in other care environments. However, it is worth noting that the majority of HIV patients in care in the US currently receive services through at least one publicly-supported program (*e.g.*, Ryan White Program, Medicaid, Medicare), which enhances the potential external validity of the findings. Second, the data are focused on efforts pre-implementation of the HIEs. Thus, they speak to the processes of building stakeholder trust and buy-in, but do not reflect on satisfaction or efficacy of the systems once in use. Third, the data collected with patients was limited to quantitative surveys. This curtails our ability to explore in detail the perspectives that drove those patients’ attitudes. In collaboration with the six HIE sites and based on our resources, we decided not to include patients in the qualitative sample. Interviewing them pre-implementation may not have yielded much specific information, but rather more potential scenarios, about the HIEs they were not familiar with. Providers were able to offer insights into some of the factors that likely affect patient attitudes. Given the many parallels between provider and patient attitudes and given that patients learn much of their information about systems from providers; we believe that those provider insights are relevant. They cannot, however, be considered definitive evidence about patients’ experiences with IT. Similarly, it is beyond the purpose of this paper to examine specific concerns of groups of patients. However, our patient quantitative data do not seem to indicate a difference according to ethnicity, gender, or sexual orientation in relationship to data sharing. Fourth, we did not interview institutional leaders outside the projects, who were more distal players within the partnering institutions. That was not part of our work, as we were tasked with evaluating the specific projects. Thus, we are not representing their personal opinions, but rather the institutional responses to the proposed projects based on the perceptions of project staff and providers. They offered good insights into the kinds of barriers at the institutional level, but those reports may not offer the same degree of nuance that would be found by interviewing those distal players.

## **Conclusions**

HIEs are an important component of HIT. They may help improve coordination and efficiency of care across medical clinics, support service agencies, payers, and public health entities. Providers and other stakeholders are willing to accept new technologies to promote more rapid and effective data sharing even when care involves a disease that historically has been seen as highly stigmatized. However, this acceptability appears to be dependent on the time and effort expended to understand and address potential concerns related to data sharing and confidentiality, and to fully educate and build trust among stakeholders about the nature of the new systems and how they will be used.

## **Competing interests**

The authors declare that they have no competing interests.

## **Authors’ contributions**

AM collected, analyzed and interpreted the data, conceptualized and drafted the manuscript; WS conceived of the design of the study, conceptualized and drafted the manuscript; KK collected, analyzed and interpreted the data, and helped to conceptualize and draft the manuscript; CP analyzed the data and helped to draft the manuscript; SS helped to conceptualize and draft the manuscript; DC analyzed the quantitative data and helped to conceptualize and draft the manuscript; JM conceived of the design of the study and helped to conceptualize, draft, and review the manuscript. All authors read and approved the final manuscript.
